# Serum cortisol level as marker of stress in camels: relationship with immunological profile

**DOI:** 10.3389/fvets.2025.1570564

**Published:** 2025-03-25

**Authors:** Jamal Hussen, Hind Althagafi

**Affiliations:** ^1^Department of Microbiology, College of Veterinary Medicine, King Faisal University, Al-Ahsa, Saudi Arabia; ^2^Department of Biology, College of Science, Princess Nourah Bint Abdulrahman University, Riyadh, Saudi Arabia

**Keywords:** camel, stress, cortisol, leukocytes, immunophenotype

## Abstract

The present study evaluated serum cortisol levels as a stress indicator in camels and analyzed the influence of some physiological and pathological factors on cortisol levels and their relationship with immunological parameters. A total number of 169 camels (*Camelus dromedarius*) were grouped in a healthy group (*n* = 106 camels), a slaughterhouse group (*n* = 20 camels), a Surra-affected group (*n* = 27 camels), and a metritis-affected group (*n* = 16 camels). Female camels exhibited higher cortisol levels compared to males, with non-pregnant and lactating she-camels showing elevated levels relative to their pregnant and non-lactating counterparts. No significant differences in cortisol levels were observed among camel breeds (Majaheem, Magateer, Sawahli, and Omani). Elevated cortisol levels were observed in stressed camels (pre-slaughter) and diseased camels, including those with Surra and bacterial metritis, confirming the reliability of cortisol as a stress marker in this species. Increased cortisol levels were associated with leukocytosis, neutrophilia, and a higher neutrophil-to-lymphocyte ratio. Phenotypically, elevated cortisol level was associated with an expanded CD4 T-cell population, reduced γδ T cells percentage, decreased CD172a expression on neutrophils and monocytes, reduced CD14 and CD163 expression on monocytes, and enhanced CD45 and MHC I expression on lymphocytes. Functionally, higher cortisol levels were linked to increased reactive oxygen species (ROS) production in blood phagocytes. These findings highlight the modulatory effects of cortisol on the camel immune system and emphasize the importance of considering gender and reproductive status when evaluating stress in camels.

## 1 Introduction

Early detection of stress is one of the most effective strategies to counteract stress-induced disorders in humans and animals. Various markers have been identified as potent indicators of stress including endocrine changes leading to elevated levels of hormones, changes in blood levels of heat shock proteins, acute phase proteins, lipid peroxidation markers, and microRNAs (1, 2). High circulating level of cortisol has been identified as one of the most reliable indicators of stress in several species (1, 3).

The most common factors that are responsible for stress in livestock include road transportation, type of the production and housing system (extensive or intensive, social mixing or regrouping of animals), and environmental stress such as heat stress (3–9). In addition, several physiological factors such as breed, gender, age, pregnancy, lactation, and parturition were found associated with variations in serum cortisol levels in different species (10–14).

In camels, Saeb et al. reported a serum cortisol level between 26 and 40 nmol/L (9.4 and 14.5 ng/mL) in healthy animals (15). Other studies, however, reported lower concentration (3.8 nmol/L) of plasma cortisol in healthy camels (16). Elevated cortisol levels have been found indicative for different stressful situations in camels including road transportation and long-term dehydration (13, 15–17). Animal management practices at slaughterhouses, such as type and duration of animals loading and transportation are among the stressful situations resulting in increased serum cortisol levels in camels.

Stress response in animals is regulated by the hypothalamic–pituitary–adrenal (HPA) axis that regulates the secretion of cortisol (18). Stress-induced activation of HPA axis results in the release of corticotrophin-releasing factor by hypothalamus which stimulates the adenohypophysis to secrete adrenocorticotropic hormone (ACTH) in blood circulation which in turn stimulates the secretion of cortisol by the adrenal gland. As a result, the immune system gets sensitized and responds immediately to the peripheral stress through several changes in humoral and cellular immune parameters (19). Studies in cattle reported a marked stress-associated leukocytosis and neutrophilia with a decrease in lymphocyte numbers and shifts in their composition (20). Especially, the decrease in T cell numbers with their reduced blastogenic and cytokine production response have been linked to higher susceptibility to infectious diseases in stressed cattle (4, 20, 21).

Studies on stress indicators in camels and their immune response to stress are scarce. The present study was, therefore, undertaken to investigate the impact of selected physiological and pathological factors on serum cortisol levels in camels. In addition, the study aimed at the analysis of the association between high cortisol levels in camels and changes in some phenotypic and functional immune parameters.

## 2 Materials and methods

### 2.1 Animals

The study was conducted between July and November 2024. Some samples were, however, collected during the years 2020 to 2023. A total number of 169 camels (*Camelus dromedarius*) were involved in the present study. The animals included a healthy group (H), a slaughterhouse group (SL), a Surra group (S), and a metritis group (ME). The healthy group included 106 clinically healthy camels including 88 female and 18 male camels aged between 0.5 and 13 years (mean age 6.6 years). The female camel group contained 30 non-pregnant she-camels and 58 pregnant she-camels from the 3^rd^ to 7^th^ month. The camels were selected from different private camel farms in the eastern and middle region of Saudi Arabia as healthy animals without any signs of disease based on clinical examination by a trained veterinarian. The slaughterhouse group included 20 clinically healthy male camels (2 years old) selected from the animals that were admitted for normal slaughtering at Al-Omran Slaughterhouse (Eastern Province, Saudi Arabia). The Surra-affected camel group included 27 animals (23 females and 4 males) aged between 2 and 15 years (mean age 5.8 years). The clinical metritis group included 16 she-camels aged between 6 and 16 years (mean age 10.3 years). The serum samples related to the Surra-affected and the metritis-affected groups were from two previously published studies (22, 23), and were retrospectively analyzed for serum cortisol levels. Detailed information for animal age, breed, gender, lactation and pregnancy status are described in (Supplementary Table 1). All farms included in the study used similar nutritional and production practices. For all farms, camels were kept under a traditional management system, where they grazed during the daytime. At night, the camels were housed in a fen barn and fed on hay and barley in addition to bread and dates with water provided ad libitum.

### 2.2 Blood collection and separation of serum

Blood samples were collected from the jugular vein in EDTA-containing tubes (for cellular analysis) or serum collection tubes (for cortisol measurement) and transported within 1–2 h to the laboratory. All tubes were from Guangzhou Improve Medical Instruments Co., Ltd. (Guangzhou, China). Serum collection was performed by centrifugation of blood at 1,000 × *g* for 15 min at room temperature. Blood sampling from the healthy and slaughterhouse groups was performed between July and October 2024 and separated serum samples were stored at −80°C until analysis. For the metritis- and Surra-affected animals, blood samples were collected between January and June from 2020 to 2023 and separated serum samples were stored at −80°C until analysis. For all animals, the blood sampling time was during the morning time between 7:00 and 9:00 am.

### 2.3 Competitive enzyme-linked immunosorbent assay for the measurement of serum cortisol levels

Quantitative determination of cortisol in serum was performed using a commercial enzyme immunoassay (DRG® Cortisol ELISA Kit), according to the guidelines of the manufacturer (The, DRG, Springfield, NJ 07081 USA). For this, 20 μl serum sample was added together with 100 μl of the kit conjugate (horseradish peroxidase-conjugated cortisol) followed by incubation for 1 h at room temperature. During this time, sample Cortisol will compete with the conjugated cortisol for binding to the plate-bound antibody. After incubation, the plate was washed three times using ELISA washing buffer followed by adding 100 μl of 3,3′,5,5′-Tetramethylbenzidine (TMB) ready to use substrate and chromogen solution. After 20 min of incubation at room temperature in the dark, the reaction was stopped by the addition of 100 μl of the stop solution (0.16 M H_2_SO_4_). Finally, the optical density values, which are inversely proportional to the concentration of Cortisol in the sample, were measured using ELISA reader (iMark Bio-Rad laboratories). For each plate, a standard curve was prepared from different standard Cortisol concentrations. The assay has a sensitivity of 1.0 ng/ml (6.0 nmo/L) and a detection range between 1.3 and 800 ng/ml. Sample Cortisol concentrations were calculated using a standard semi-logarithmic curve fit.

### 2.4 Cell separation for flow cytometry

Blood leukocytes were separated by hypotonic lysis of erythrocytes followed by centrifugation as previously described. Briefly, EDTA blood (1 ml) was incubated for 20 s with 5 ml distilled water in a 15 ml sterile falcon tube to induce red blood cell lysis. Subsequently, tonicity was restored by the addition of 5 ml 2× PBS, and the tube was centrifuged at 3000 RPM for 10 min at 10°C. For complete removal of red blood cells, the lysis step was repeated twice followed by centrifugation at 2,200 and 1,500 RPM for 10 min each round. Finally, the pellet was resuspended in cold PBS and adjusted to 1 × 10^6^ cell/ml. Cell vitality was measured by the addition of propidium iodide and was always above 93%.

### 2.5 Flow cytometric analysis of leukocyte subsets

Separated leukocytes were labeled with monoclonal antibodies (mAbs) to the leukocyte antigens CD45, CD44, CD11a, major histocompatibility (MHC) class-I, MHC class II, BAQ44A (B cell), WC1 (γδ T cell), CD4 (helper T cell), CD172a, CD14, and CD163 (24, 25). In the first staining step, cells (1 × 10^6^ cell/well) were incubated in a 96-well plate with the primary mAbs for 15 min at 4°C. After washing with PBS/BSA buffer, the second staining step was done by incubating the cells with fluorochrome-labeled antibodies to mouse IgM, IgG1, and IgG2a (Invitrogen). Finally, the cells were washed and analyzed by flow cytometry (Becton Dickinson Accuri C6 flow cytometer; Becton Dickinson Biosciences, San Jose, California, USA).

### 2.6 Analysis of reactive oxygen species level in neutrophils and monocytes

The level of reactive oxygen species (ROS) in camel neutrophils and monocytes was analyzed using the ROS-dye dehydrorohdamin-123 (DHR-123) and flow cytometry. Leukocytes (1 × 10^6^ in 100 μl RPMI medium) were loaded with DHR-123 for 30 min at 37°C and 5% CO_2_. Stained cells were analyzed by flow cytometry (Accuri C6 flow cytometer; BD Biosciences). Baseline ROS level was measured based on the increase in the green fluorescence (mean FL-1) of neutrophils and monocytes.

### 2.7 Statistical analysis

Means and standard error of the mean (SEM) were calculated using the column statistic function of the Prism software (GraphPad). Data normality was calculated using the Shapiro–Wilk test. The comparison between the four camel breeds was performed using the Kruskal-Wallis one-way analysis-of-variance (ANOVA) in combination with Dunn’s *post hoc* test. The comparison between values based on pregnancy and lactation was done using unpaired student’s t test. The impact of gender on serum cortisol level was statistically analyzed using Mann–Whitney U test (not normally distributed values). In addition, the Common Language Effect Size (CLES) was calculated based on *Cohen’s d* using an online calculation tool^1^ and was presented alongside the *p*-values. Although both male and female camels from different ages and breeds were represented in the population of the studied camels, which was available for the study, the lack of power analysis to determine sample size at the beginning of the study is a limitation of the present study.

## 3 Results

### 3.1 Impact of breed, gender, age, pregnancy, and lactation status on cortisol levels in healthy camels

**Figure 1:**
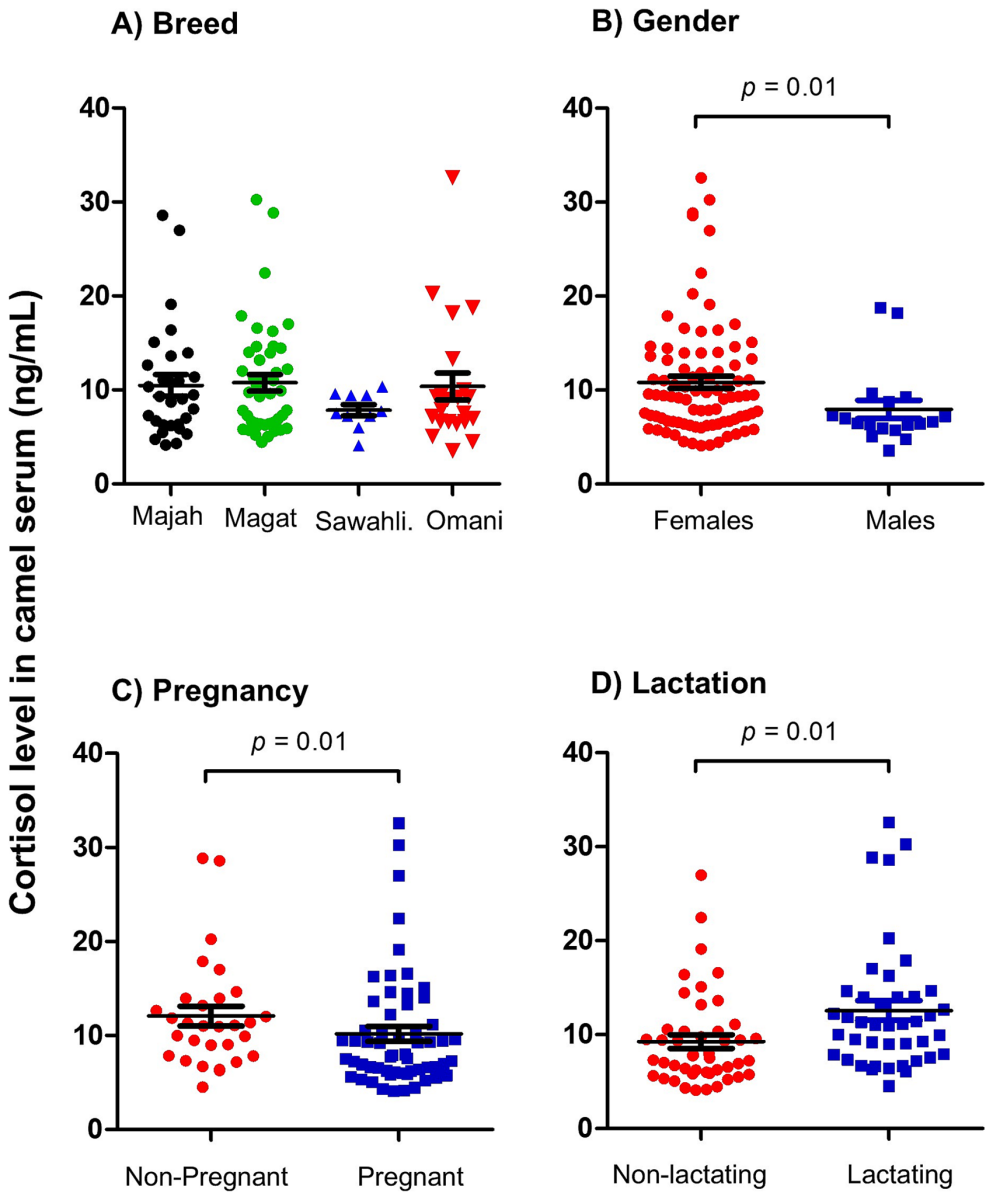
Analysis of serum cortisol levels. Serum samples were collected from healthy camels and analyzed for cortisol using competitive ELISA. Cortisol concentration in camel serum was calculated based on the standard curve generated using standard cortisol concentrations. Cortisol levels were presented as scattered dot plot graph based on camel breed **(A)**, camel gender **(B)**, pregnancy **(C)**, and lactation **(D)** status. The comparison between the four camel breeds was performed using the one-way analysis of variance (ANOVA). The comparison between values based on pregnancy and lactation was done using student’s *t* test. The impact of gender was statistically analyzed using Mann–Whitney U test.

Normal values for cortisol levels in serum of healthy camels ranged between 3.6 and 32.6 ng/ml with a mean ± SEM of 10.3 ± 0.6 ng/ml (Table 1). The comparison between samples collected from four camel breeds revealed no significant differences (*p* > 0.05) between cortisol levels in healthy Majaheem, Magateer, Sawahli, and Omani camels (Figure 1A). Female camels (10.8 ± 0.6 ng/ml) showed significantly (*p* = 0.01; CLES = 0.64) higher cortisol levels in their serum than males (7.9 ± 0.9 ng/ml) (Figure 1B). Within the female camels, the comparison between pregnant and non-pregnant she-camels (Figure 1C) revealed significantly higher cortisol levels in serum of non-pregnant (12.1 ± 1.0) than pregnant (10.2 ± 0.8) she-camels (*p* = 0.01; CLES = 0.58). In addition, lactating she-camels (12.5 ± 1.0 ng/ml) had significantly (*p* = 0.01; CLES = 0.65) higher cortisol levels than non-lactating (9.2 ± 0.7 ng/ml) she-camels (Figure 1D). Furthermore, correlation analysis revealed no significant (*p* > 0.05) association (r = 0.184) between animal age and cortisol level (data not shown).

**Table 1:** Serum cortisol levels (ng/ml) in healthy camels.

		Number	Cortisol (Mean ± SEM)
All camels		106	10.3 ± 0.6
Breed	Majaheem	29	10.5 ± 1.1
	Magateer	45	10.7 ± 0.8
	Sawahli	10	7.8 ± 0.6
	Omani	22	10.3 ± 1.4
Gender^*^	Female	88	10.8 ± 0.6
	Male	18	7.9 ± 0.9
Pregnancy^*^	Non-pregnant	30	12.1 ± 1.0
	Pregnant	58	10.2 ± 0.8
Lactation^*^	Non-lactating	46	9.2 ± 0.7
	Lactating	42	12.5 ± 1.0

* indicates significant effect of the parameter on serum cortisol level.

### 3.2 Camels at slaughterhouse showed elevated cortisol levels in their serum

**Figure 2:**
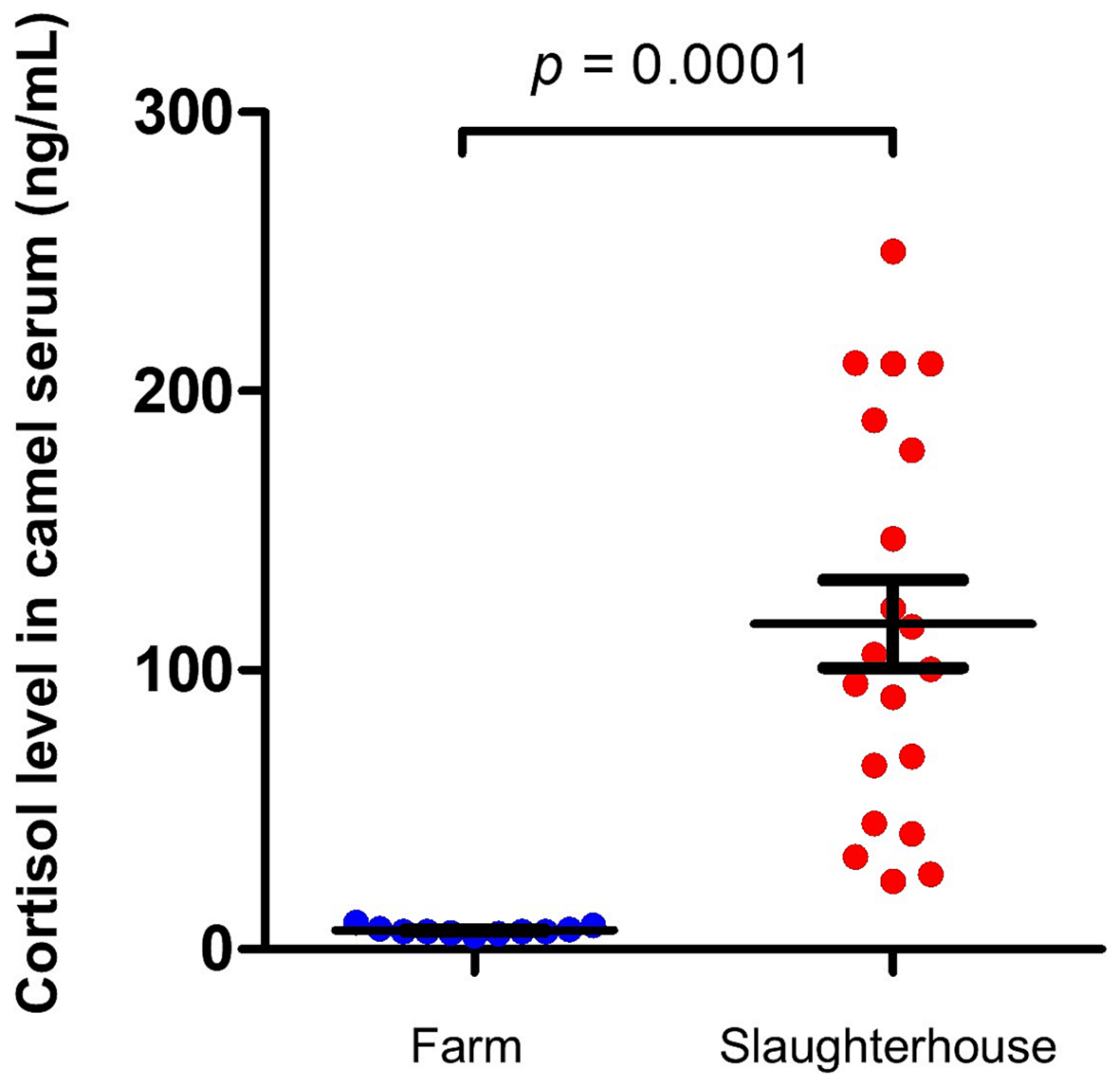
Impact of slaughterhouse stress on serum cortisol levels in camels. Serum samples were collected from camels at slaughterhouse (*n* = 20) and camels on their farms (*n* = 11) and analyzed for cortisol using competitive ELISA. Cortisol levels were calculated and presented as scattered dot plot graph. *p* values were calculated using student’s *t* test.

To avoid interaction with a possible impact of camel gender on the results and as all camels in the slaughterhouse group were male camels, we compared this group with male camels from the healthy group. Serum samples collected from camels at slaughterhouse directly before slaughtering showed increased cortisol levels (116.6 ± 15.8 ng/ml) which were 10 times higher (*p* = 0.0001; CLES = 0.92) than cortisol levels in serum samples collected from male camels on their farms (6.8 ± 0.4 ng/ml) (Figure 2).

### 3.3 Impact of disease on serum cortisol levels in camel

**Figure 3:**
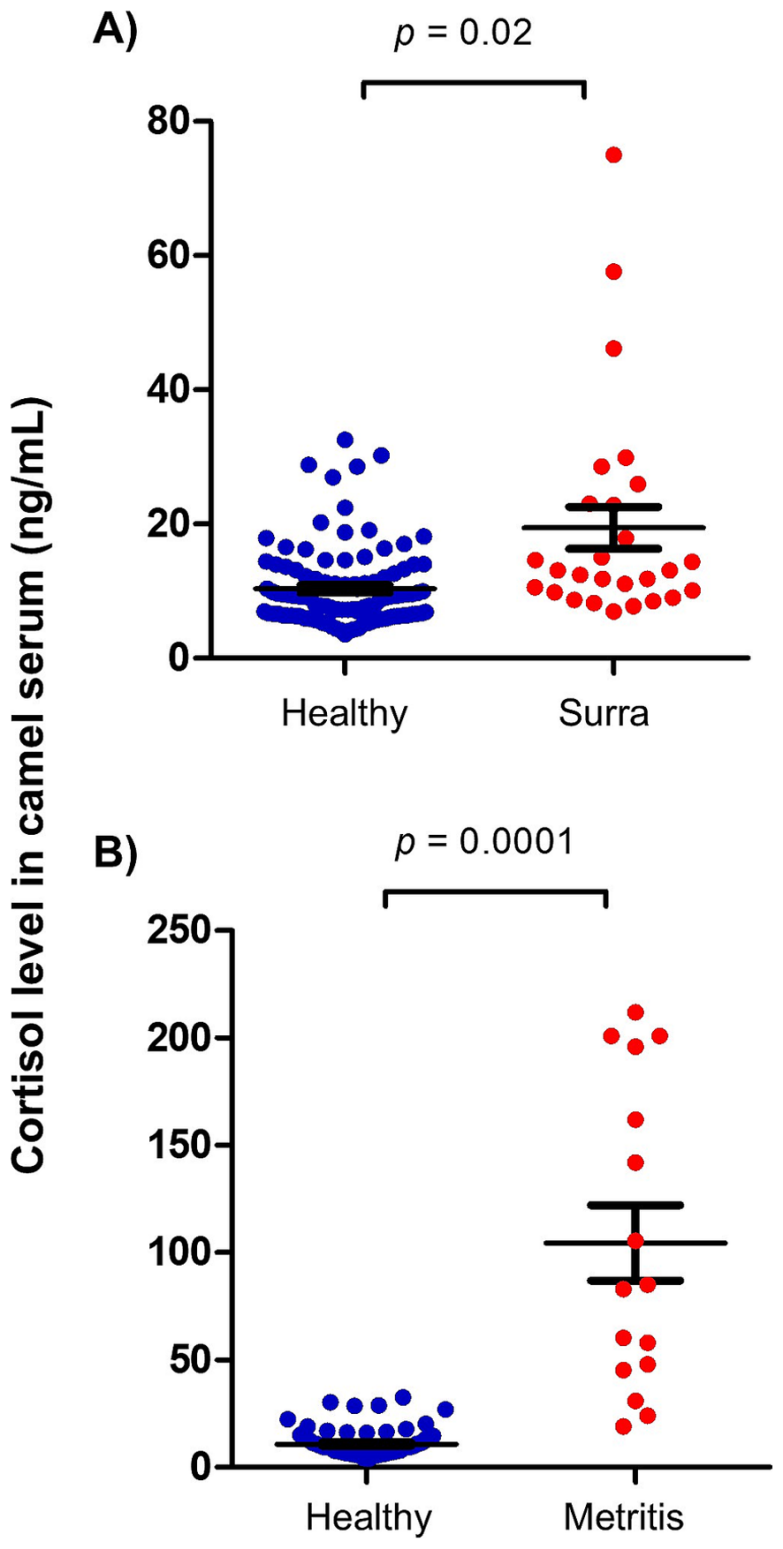
Impact of disease on serum cortisol levels in camels. Serum samples were collected from **(A)** surra-affected camels (*n* = 27), **(B)** camels with metritis (*n* = 16), and healthy camels (*n* = 106) and analyzed for cortisol using competitive ELISA. Cortisol levels were calculated and presented as scattered dot plot graph. *p* values were calculated using student’s *t* test.

To evaluate the impact of infectious diseases on blood cortisol levels in camels, serum samples previously collected from camels affected by Surra and camels with bacterial metritis were retrospectively analyzed for cortisol levels. The results indicate significantly elevated cortisol levels in blood of diseased animals. Cortisol concentration in serum of Surra-affected camels (19.4 ± 3.1 ng/ml) was about two times higher than healthy (10.3 ± 0.6 ng/ml) camels (*p* = 0.02; CLES = 0.76) (Figure 3A). Similarly, she-camels with metritis (104.6 ± 17.7 ng/ml) had 10 times higher cortisol in their serum compared to healthy she-camels (10.8 ± 0.6 ng/ml) (*p* = 0.0001; CLES = 0.99) (Figure 3B).

### 3.4 Association between selected immune parameters and serum cortisol levels in camel

The correlation results between selected immune parameters and serum cortisol levels in camels are shown in Table 2. The total number of leukocytes (r = 0.56) in camel blood, the number of neutrophils (r = 0.52) and the neutrophil to lymphocyte ratio (r = 0.32) were positively correlated with serum cortisol levels (*p* < 0.05). Within blood lymphocytes, a negative correlation was found between the percentage of gd T cells (r = −0.36) and cortisol level, while the number of CD4+ T cells (0.39) was positively correlated with cortisol level. Phenotypic analysis of neutrophils revealed negative correlation (r = −0.51) between CD172a expression and cortisol level. For lymphocytes, the expression density of CD45 (r = −0.30) and MHC class I (r = −0.39) molecules was positively correlated with serum cortisol level. The abundance of the monocyte markers CD14 (r = −0.40), CD163 (r = −0.47), and CD172a (r = −0.51) was negatively correlated with serum cortisol level. On the other hand, a positive correlation was identified between reactive oxygen species level in neutrophils (r = 0.67) and monocytes (r = 0.69) and serum cortisol level (Table 2).

**Table 2:** The correlation results between selected immune parameters and serum cortisol levels in camels.

	Parameter	Number of XY pairs	Spearman r	*p* value (two-tailed)	Is the correlation significant?
Leukocyte composition	WBC (10^3^Cell/μl)	43	0.5461	**0.0002**	**Yes**
	Neutrophils (%)	43	0.2555	0.0982	No
	Eosinophils (%)	43	0.01997	0.8988	No
	Lymphocytes (%)	43	−0.2736	0.0758	No
	Monocytes (%)	43	−0.02858	0.8556	No
	Neutrophils (cell/μl)	43	0.5256	**0.0003**	**Yes**
	N/L ratio	43	0.322	**0.0352**	**Yes**
	Eosinophils (cell/μl)	43	0.2319	0.1345	No
	Lymphocytes (cell/μl)	43	0.183	0.24	No
	Monocytes (cell/μl)	43	0.417	0.0054	**Yes**
Lymphocyte composition	CD4+ (% of Ly)	43	0.1956	0.2087	No
	WC1 (% of Ly)	43	−0.3627	**0.0168**	**Yes**
	B cell (% of Ly)	43	−0.2037	0.1901	No
	CD4 (cell/μl)	43	0.3963	**0.0085**	**Yes**
	WC1 (cell/μl)	43	−0.2441	0.1147	No
	B cell (cell/μl)	43	0.006408	0.9675	No
Neutrophil phenotype (MFI)	CD45	36	0.06918	0.6885	No
	CD44	36	0.1381	0.4218	No
	CD11a	20	−0.08481	0.7222	No
	MHC-I	20	−0.09168	0.7007	No
	CD14	36	0.2039	0.2329	No
	MHC-II	36	−0.1581	0.3571	No
	CD172a	36	−0.5133	**0.0014**	**Yes**
Lymphocyte phenotype (MFI)	CD45	41	0.3094	**0.049**	**Yes**
	CD44	43	0.01062	0.9461	No
	CD11a	27	0.2378	0.2324	No
	MHC-I	27	0.3948	**0.0415**	**Yes**
Monocyte phenotype (MFI)	CD45	43	0.1649	0.2906	No
	CD44	43	0.2327	0.1332	No
	CD11a	27	0.1414	0.4818	No
	MHC-I	27	0.3696	0.0578	No
	CD14	43	−0.4022	**0.0075**	**Yes**
	MHC-II	43	0.008947	0.9546	No
	CD163	43	−0.4788	**0.0012**	**Yes**
	CD172a	43	−0.5168	**0.0004**	**Yes**
ROS production	Neutrophils	29	0.6788	**< 0.0001**	**Yes**
	Monocytes	29	0.6935	**< 0.0001**	**Yes**

WBC: white blood cells; CD: cluster of differentiation; MFI: mean fluorescence intensity; WC1: workshop cluster 1; ROS: reactive oxygen species. Bold values indicate significant correlation between the parameter and cortisol level in camel serum.

## 4 Discussion

The employment of various indicators in the early detection of stress is one of the most effective strategies to counteract stress-induced disorders in humans and animals. Recent reports support the role of early stress detection in preventing systemic complications (26). The present study aimed at the evaluation of serum cortisol level as an indicator of stress in camels. For this, cortisol levels were retrospectively measured in serum collected from camels at slaughterhouse, from *trypanosoma*-affected camels, and from camels with bacterial metritis and compared it with normal cortisol levels in healthy camels. In addition, the impact of selected physiological factors on serum cortisol levels in healthy camels was investigated.

The comparison between blood cortisol levels in Majaheem, Magateer, Sawahli, and Omani camels indicates no impact of camel breed on blood cortisol level. As the animals involved in the present study were healthy non-stressed animals, the conclusion that breed does not influence cortisol levels in camels requires validation under stress-inducing circumstances, necessitating further research to evaluate breed-specific cortisol responses after exposing camels from different breeds to stressful stimuli (road transportation, heat stress, housing stress). Few studies investigated the impact of age, gender, and reproductive status of animal on serum cortisol levels in camel. Although the results of the present study are in agreement with the study of Sazmand et al., who reported no association between camel age and serum cortisol level, they, however, contrast the same study regarding the observed higher cortisol concentration in female than male camels (13). Similarly, the results of the present study are in contrast to a recent study by Mohamed et al., which reported the association between pregnancy and increased serum cortisol levels in camels (27). Pregnancy-associated change in serum cortisol levels was recently reported for the donkey species with increased cortisol levels during late pregnancy (28). Given that all the pregnant she-camels in the present study were in their early to mid-pregnancy, further studies should follow up the changes in serum cortisol levels during the whole pregnancy period in camels. In agreement with our study, Mohamed et al. reported no impact of camel lactation status on serum cortisol concentration. Further studies with higher numbers of camels are required to see whether the observed differences between the present study and previous studies are related to different analytic methodologies. Additionally, the impact of several factors including environmental conditions, animal nutrition, and management practices on cortisol levels in camels were investigated in several studies (29–31). In the present study, although the homogeneity in production systems and nutritional practices in the camel farms located in this geographical area (Eastern of Saudi Arabia) reduces the probability that these factors would affect our results, we cannot exclude a possible impact of these factors on the measured cortisol levels in camel serum. Furthermore, several studies reported seasonal variation in cortisol levels and the circadian regulation and diurnal secretion of cortisol leading to changes in serum cortisol levels (32–35). In the present study, although blood samples were collected during the morning time (between 7:00 and 9:00 am), we cannot exclude the possible impact of this minimal variation in sample collection time or a possible seasonal effect on the obtained cortisol levels, presenting an additional limitation of the study.

A wide range of values were reported in the scientific literature for cortisol concentration in serum of healthy camels. Although serum cortisol concentrations measured in healthy camels in the present study are in line with values reported for camels by several authors (15, 17, 36, 37), lower as well as higher levels were also reported in the literature (27, 31, 38). Radioimmunoassay and enzyme immunoassays are the main analytic methods used by most studies to measure cortisol levels in camel serum or plasma. Whether different analytic methods that were employed for cortisol measurement may have contributed to the variation in the reported ranges, is still to be investigated.

The response of camels to stressful situations has been investigated in some studies. In a recent study, radioimmunoassay was employed to measure cortisol levels in serum samples collected from 50 male dromedary camels at slaughterhouse. The study reported high (80.2–107.2 ng/ml) to very high (133.7–198.0 ng/ml) levels of serum cortisol in 26 camels (about 50% of the animals), while the other 24 camels showed low serum cortisol levels (13.0–67.9 ng/ml) (36). Similarly, Sayah et al. (39) measured high to very high serum cortisol levels in male Sahraoui camels at slaughterhouse. The study reported higher cortisol levels in animals held in lairage for short period (874 ± 631 ng/ml) after arrival and before slaughter (1 to 8 h) than in animals held for longer time (48–96 h) (127 ± 39 ng/ml). Although the present study confirms findings of previous studies on the impact of pre-slaughter handling on stress in camels (17, 36, 39), it could not specifically identify the exact factors responsible for the increased cortisol levels at slaughterhouse. Based on other studies in camels, common factors with major responsibility for elevated blood cortisol in pre-slaughtered camels mainly include the duration of pre-slaughter operations, the time until loading of the animals, unloading, water and food deprivation, and the transportation to the slaughterhouse (15, 17). The reliability of blood cortisol level as an effective indicator of stress in camels was proven in numerous studies (15, 36). In the present study, the elevated concentrations of serum cortisol in camels at slaughterhouse and in diseased camels (Surra and metritis) is in support of this. In addition, the observed differences in cortisol levels between males and females, pregnant and non-pregnant camels, and lactating and non-lactating camels confirm previous findings reporting that physiological parameters, such as gender and reproductive status of the animal, are within the factors that largely affect the animal response to stress (10–12).

The immune response to stress has been characterized in several species including cattle (40), sheep (19) rats (41), zebrafish (42), and human (43). Such studies are still lacking in camels. In the present study, higher cortisol levels were found associated with significant changes in some phenotypic and functional parameters of the camel immune system. Due to the lack of previous studies in camels, the changes observed in the present study were compared with findings in other species. The observed association between elevated cortisol levels in camels and increased numbers of total leukocytes and neutrophils as well as the higher neutrophil to lymphocyte ratio is in agreement with a typical stress leukogram pattern characterized by leukocytosis and neutrophilia (42, 43), while the positive correlation between cortisol level and the number of CD4+ T cells seems in contrast to the well-known stress-associated lymphopenia with reduced numbers of T helper cells. Studies in several species showed that glucocorticosteroid-induced leukocytosis is a result of demargination of neutrophils (shifting of marginal neutrophils to the circulation) due to modulation of cell adhesion molecules (44). Although the observed association between high cortisol levels and lower expression of CD172a on neutrophils, a signal-regulatory protein alpha (SIRPα) with essential role in adhesion and trans-endothelial migration of neutrophils (45), supports this finding, further studies are required to explore the exact mechanisms behind the changes in camel leukogram associated with elevated cortisol levels.

The impact of stress on blood monocytes was investigated in many species (46). The observed negative correlation between the abundance of the LPS receptor CD14, the scavenger receptor CD163, and the signal regulatory protein (SIRP-alpha) CD172a on monocytes and serum cortisol level supports a stress-induced alteration in monocyte phenotype. Together with toll-like receptor (TLR)-4, CD14 plays an essential role in the recognition of the cell wall of gram-negative bacteria by monocytes and macrophages. The best characterized function of CD163 is related to the binding of Hemoglobin:Haptoglobin complexes playing a role in tissue homeostasis (47). On the other hand, CD172a is known for its regulatory role in immune function by binding to its ligand CD47 (48). Given the different roles of these surface molecules during the immune response, the observed correlations indicate the complexity of the relationship between cortisol on monocyte biology. While reduced CD14 expression in animals with higher cortisol levels suggests reduced innate immune sensing of pathogens by monocytes, downregulation of CD163 and CD172a indicates negative impact of cortisol on regulatory mechanisms responsible for resolution of inflammation. Therefore, further studies are required to explore how those phenotypic alterations are reflected on monocyte function in stressed camels.

Innate immune cells such as neutrophils and monocytes produce reactive oxidative species and free radicals upon activation. Enhanced ROS production has been found associated with an inflammatory response with tissue injury and the release of danger signals (DAMPs) such as s100 proteins (49). In the present study, the association between higher reactive oxygen species levels in neutrophils and monocytes and serum cortisol levels reflects an oxidative stress response in immune cells from stressed camels.

As reported in the scientific literature, the stability of cortisol concentrations in saliva samples after 6 years of storage at −80°C and in serum samples after 3 to 40 years of storage at −20 (50, 51), it is unlikely that obtained serum cortisol results were affected by the long time storage (from 2020 for the metritis group and 2022 for the Surra group).

## 5 Conclusion

Increased cortisol levels in pre-slaughtering camels, Surra-diseased camels, and camels with bacterial metritis confirm the reliability of serum cortisol as stress indicator in this species. The variation in cortisol levels according to gender and reproductive status of the animal indicates the importance of considering these factors when evaluating the stress situation in camels. The observed fluctuations in the numbers of immune cell subsets and the changes in their phenotype may affect their functions, thus affecting the animal’s resistance to infectious diseases. Future research should, therefore, address the relationship between stress and the functional capacity of the immune system in camels.

## Data Availability

The original contributions presented in the study are included in the article/Supplementary material, further inquiries can be directed to the corresponding author.

## Footnotes

^1^ https://www.psychometrica.de/effect_size.html
